# Adiponectin Influences FGF2 in the Developing Porcine Corpus Luteum

**DOI:** 10.3390/vetsci9020077

**Published:** 2022-02-12

**Authors:** Rita Flores, Martha Ramirez, Luis Ayala, Elizabeth A. Benavides, Fang Xie, Adrian Aaron Arellano, Randy Louis Stanko, Michelle Renee Garcia

**Affiliations:** 1 Department of Biochemistry and Molecular Biology, Oklahoma State University, Stillwater, OK 74078, USA; ritaaf@ostatemail.okstate.edu; 2 Department of Animal Science and Veterinary Technology, Texas A&M University-Kingsville, Kingsville, TX 78363, USA; martita_4@msn.com (M.R.); layala.hilltop@gmail.com (L.A.); randy.stanko@tamuk.edu (R.L.S.); 3 Department of Agricultural Sciences, Texas State University, San Marcos, TX 78666, USA; eab145@txstate.edu; 4 Department of Surgery, University of California-San Francisco, San Francisco, CA 94142, USA; fang.xie2@ucsf.edu; 5 College of Veterinary Medicine, College Station, Texas A&M University, Corpus Christi, TX 77843, USA; aaarellano@cvm.tamu.edu

**Keywords:** adiponectin, FGF2, AdipoR2, CL, porcine

## Abstract

Luteal angiogenesis is regulated by pro-angiogenic hormones including fibroblast growth factor 2 (FGF2) and angiopoietin 1 (Ang1), which are regulated by the adipokine leptin during development. Another adipokine, adiponectin, exhibits an inverse relationship with leptin and has been identified in the CL. Therefore, it is hypothesized that adiponectin will influence pro-angiogenic hormones in the developing porcine CL. Crossbred sows were randomly allocated to one of two days of the estrous cycle, day 5 (D5; *n* = 4) or day 7 (D7; *n* = 5) for CL collection. Tissue was processed for immunohistochemical localization of adiponectin receptor 2 (AdipoR2), gene expression of FGF2, Ang1, leptin, AdipoR2, and cell culture for adiponectin treatment. The expression of AdipoR2 tended (*p* = 0.09) to be higher in D7 lutea and was more prevalently localized to the cell surface of large and small luteal cells than in D5 tissue. Adiponectin influenced (*p* ≤ 0.05) FGF2, leptin, and AdipoR2 gene expression relative to the dose and day (D5 or D7). Collectively, the evidence supports the supposition that adiponectin influences angiogenic factors in the developing CL.

## 1. Introduction

The corpus luteum (CL) is a highly vascular ovarian structure formed from an ovulated Graafian follicle that is essential for pregnancy maintenance. This morphological and biochemical transformation of follicular cells into small and large luteal cells is supported by pre-existing vasculature that ramps up angiogenic activity to sustain rapid luteal development [1]. Luteal expression of angiogenic regulators such as the vascular endothelial growth factor (VEGF), fibroblast growth factor 2 (FGF2), and angiopoietin 1 (Ang1) differ relative to the stage (early, mid, and late) of the luteal phase of the estrous cycle to initiate and maintain vascularization (angiogenic) processes [1,2,3]. However, FGF2 appears to exhibit a greater influence on luteal angiogenesis than VEGF [1,3]. Moreover, in the porcine CL, only FGF2 gene expression exhibits significant daily changes throughout development, increasing on days 4 and 6 and decreasing on days 5 and 7 in the early stage of the luteal phase [4]. The daily expression of FGF2 in the developing CL has not been reported in other species; however, the anti-angiogenic glycoprotein thrombospondin exhibits a similar pattern of expression in granulosa cells of antral follicles [5]. This suggests that ovarian angiogenesis may be a precisely timed mechanism associated with the regulation of growth and developmental processes. Leptin is a potent adipokine with angiogenic properties [6] that regulates the expression of FGF2 and Ang1 in dispersed lutea cell cultures, specifically decreasing FGF2 in cells obtained from D7 lutea [4]. Both leptin and its receptor are expressed in the bovine, caprine, and porcine CL [4,7,8], but changes relative to the day of development have not been detected despite a reported association with luteal angiogenesis [7,9]. Interestingly, another adipokine, adiponectin, is also expressed in the porcine CL and influences luteal function [10]. Furthermore, it is well-documented that adiponectin often exhibits an inverse and antagonistic relationship with leptin in vascular-related processes [11].

Adiponectin is a protein structurally comprised of a globular head and collagenous stalk [12] that exits in three distinct multimeric forms, low, middle, and high molecular weight complex (LMW, MMW, HMW), which influences blood plasma half-life and subsequent bioactivity [13]. All forms have been identified in the serum of pigs [14] and all forms bind adiponectin receptors with varying affinity to activate several signaling cascades that regulate numerous processes including fatty acid oxidation and inflammation [15]. Additionally, adiponectin is associated with angiogenic mechanisms including the suppression of nascent vascular growth [16] and reduced FGF2 in non-ovarian tissues of adiponectin KO mice [17]. Such variation in biological activity is also attributed to the different adiponectin receptor isoforms, 1 and 2 (AdipoR1 and AdipoR2; 14). Both isoforms are implicated in fatty acid metabolism and steroidogenic cellular processes [18,19], but angiogenic mechanisms appear to be largely attributed to AdipoR2. Revascularization of damaged femoral arteries occurs in AdipoR1 KO, adiponectin-treated mice but is significantly attenuated in the AdipoR2 KO [20]. Furthermore, AdipoR2 mediates cellular action through PPARα activation [21] and the MAPK cascade [22], which are mechanisms that regulate Ang1 and FGF2 gene expression, respectively [23,24]. Both receptor isoforms have been identified in the porcine and bovine CL throughout the luteal phase [25,26], which implies a functional role for adiponectin in luteal tissue processes. In support, adiponectin has been reported to decrease luteal progesterone secretion [19,25], and microarray analysis of adiponectin-treated mid-stage luteal cells revealed changes in pro-angiogenic factors [27]. Collectively, adipokines leptin and adiponectin are involved in angiogenic processes and both hormones are expressed in the highly vascular CL. Furthermore, these adipokines exhibit an inverse relationship in the regulation of vascular-related processes. Hence, adiponectin may increase the gene expression of pro-angiogenic factors that are downregulated by leptin, which was postulated to have contributed to the reduced expression of FGF2 in the D5 and D7 porcine CL. Therefore, it is hypothesized that adiponectin will influence the gene expression of pro-angiogenic hormones in the developing porcine CL.

## 2. Materials and Methods

### 2.1. Animals

All animal-management practices and experimental procedures were previously published [4,7,9] and approved by the Texas A&M University-Kingsville (TAMUK) Institutional Animal Care and Use Committee (IACUC No. 2014-07-10). Ten mature, crossbred (Yorkshire x Hampshire), primiparous (single parity) sows, 24 ± 3.4 mos of age, weighing 152.4 ± 7.7 kg, related paternally to a Yorkshire sire, from the TAMUK Farm were utilized. Animals were housed in concrete pens (7 m × 13 m) in an outdoor, sheltered facility, and were fed a corn–soy-based diet (2.5 kg) once per day [28]. Sows were randomly allocated for CL tissue collection during the early stage of the luteal phase, day 5 (D5; *n* = 5) or day 7 (D7; *n* = 5), of the estrous cycle. Using an intact boar, sows were observed twice daily, morning (0700 h) and evening (1900 h), for 30 min, to detect a natural physical and behavioral estrus, i.e., lordosis response upon boar exposure, standing upon mounting, vulva swelling, the red–pink coloration of the vulva, and mucus secretion. Day 0 (D0) of the estrous cycle was determined as the day of the first estrus detection. Subsequent days of the cycle were determined to be 24 h from the initial detection of estrus, relative to morning (0700 h) or evening (1900 h) observation. Tissue collection occurred during the third consecutive estrous cycle following the observation of two consistent, consecutive estrous cycles relative to each individual sow. There were no hormonal manipulations implemented to change or control the time of estrus or the estrous cycle length. On the day of CL tissue collection, blood samples were collected, via jugular venipuncture, for analysis of serum progesterone to confirm normal luteal function.

### 2.2. CL Collection

Sows were isolated 18 h prior to tissue collection to remove potential access to feed and water was removed 12 h prior to tissue collection. Following the fasting period, animals were weighed and transported to the TAMUK surgical facility where they were pre-anesthetized using a cocktail of telazol (2.2 mg/kg) and xylazine (2.0–3.0 mg/kg) that was administered via the lateral auricular vein. After pre-anesthetic induction, animals were anesthetized using an inhalation mask placed over the external nasal nares for the administration of isoflurane gas from a vaporizer set at 4% mixed with an O_2_ flow rate of 4 L/min for initial induction to establish surgical plane anesthetization. Following the initial induction, the vaporizer was reduced to 2.5–3% with an O_2_ flow rate of 3 L/min for the surgical procedure. A vertical incision (7–10 cm) was made along the linea alba of the lower abdomen to access the reproductive tract. Ovaries were removed via blunt dissection and placed in ice-cold phenol red-free Hanks solution (Sigma Aldrich, St. Louis, MO, USA) for transportation to the laboratory and CL processing. A lutea was dissected from the ovary and divided to process for paraffin embedding, RNA extraction, and cell culture. Processed luteal tissue collected from one of the D5 sows was irreparably impaired, bringing the total number of sows in the D5 group to four total animals (D5, *n* = 4).

### 2.3. Immunohistochemistry

Luteal tissue was collected and prepared as previously reported [7]. Briefly, CL was placed in ice-cold (4 °C) fixative solution (10% formalin, 4% formaldehyde; Sigma-Aldrich) for 24 h at 4 °C on a shaker and then rinsed twice in 1× phosphate buffered saline (PBS) for 1 h. The tissue was then placed in a graded series of ethanol baths (60%, 80%, 95%, and 100% ethanol, 2× each) for 1 h at 4 °C and two separate 1 h rinses in xylene. Dehydrated CL was transferred to a paraffin (TissuePrep™; Fisher Scientific, Pittsburgh, PA, USA) bath for 2 h and then embedded in a tissue mold. The tissue was sectioned (6 μm thick each) and placed on glass slides (2 serial sections per slide; ProbeOn Plus™ slides, Fisher Scientific, San Francisco, CA, USA). The tissue was deparaffinized and rehydrated in xylene (2×) for 5 min each and rehydrated through a graded series of ethanol baths (100%, 95%, and 70%; 2× each) for 2 min each, rinsed in tap water for 5 min, and incubated in 3% (*v*/*v*) hydrogen peroxide for 10 min to block endogenous peroxidase activity. Following deparaffinization and rehydration, sections were boiled in 10 mM sodium citrate for antigen recovery, cooled to room temperature, and rinsed in the buffer (1% *w*/*v* BSA in PBS) for 5 min. Slides were processed as per the manufacturer’s recommendations using the VECTASTAIN^®^ ABC kit (Vector Laboratories, Burlingame, CA, USA). Nonspecific antibody binding was blocked by incubating the tissue in a blocking solution [1% *w*/*v* BSA and 0.3% *v*/*v* Triton-X-100 in PBS] and normal goat serum (1% *v*/*v*; Vector Laboratories) for 30 min at room temperature. Following a rinse in the buffer, slides were incubated in 3% BSA in PBS at 37 °C for 1 h and rinsed again. Slides were incubated with the AdipoR2 (1:200; rabbit host, PAA132Hu01; Cloud-Clone Corp, Katy, TX, USA) primary antibody in the buffer. For each slide incubated with the primary polyclonal antibody, a consecutive slide was incubated in normal serum (1.5% *v*/*v*) as a control. Slides were rinsed and incubated with the biotinylated secondary antibody (0.5% *v*/*v*; anti-rabbit; Vector Laboratories) for 30 min at room temperature. Following secondary antibody incubation, tissue sections were rinsed, incubated in an avidin biotinylated horseradish peroxidase complex (1% *v*/*v*) for 30 min at room temperature, rinsed, and incubated with the peroxidase substrate solution (NovaRED^®^ Substrate Kit; Vector Laboratories) for 10 min at room temperature. Each slide was stained with basic hematoxylin (Avantor/VWR, Radnor, PA, USA), rinsed, air dried, mounted in Permount™ medium (Fischer Scientific), and analyzed. Prior to primary antibody addition, a D5 and D7 tissue section were pre-incubated with 1 μg of adiponectin (HEK293; BioVender, Candler, NC) for 1 h to outcompete the antibody for AdipoR2 and confirm the specificity of the AdipoR2 antibody. Slides were observed at 20× and 40× for the location of NovaRED^®^ staining, including small (5–20 μm) and large (>20 μm) luteal cells, and vessels as previously described [29].

### 2.4. Gene Expression

Total RNA was extracted from CL tissue and cultured cells using a previously published phenol:chloroform-based procedure [7,9,30]. The gene expression of FGF2, Ang1, leptin, and AdipoR2 was analyzed with real-time PCR using the CFX96 Touch Deep Well Real-Time PCR System (Bio-Rad, Hercules, CA, USA). Before reverse transcription (RT) of mRNA, 2 μg of total RNA was treated for potential DNA contamination in a reaction mix containing moloney-murine leukemia virus reverse transcriptase (M-MLV RT), reaction buffer (50 mM Tris–HCL (pH 8.3), 75 mM KCL, 3 mM MgCl_2_, and 10 mMDTT), nucleotide mix (dATP, dCTP, dGTP, dTTP, 4 mM/μL), RQ1 DNase-1 (1 U/μL), and RNase free water in a total volume of 25 μL. Each reaction mix was incubated at 37 °C for 30 min for DNase treatment, followed by an 85 °C incubation for 10 min to terminate DNase activity. Following DNase treatment, Oligo dt15 (500 μg/mL) and M-MLV RT (200 μg/μL) were added and incubated at 37 °C for 1 h to RT mRNA. Samples were then incubated at 85 °C for 10 min to terminate the reaction. Enzymes and PCR reagents utilized for the RT were obtained from Promega (Madison, WI, USA). Transcribed cDNA was amplified by real-time PCR using primers synthesized by Integrated DNA Technologies (IDT, Coraville, IA, USA; Table 1) in a Takara SYBR^®^ Green (Madison, WI, USA) reaction mix. All PCR amplifications were conducted in duplicate for each sample. After a 10 min hot start at 94 °C, the PCR cycles were as follows: Denaturation at 94 °C for 30 sec, annealing temperature (Table 1) for 30 sec, and extension at 72 °C for 60 sec for 40 cycles, ending with a 72 °C 10 min extension [7,9]. Cyclophilin mRNA from a porcine liver was used to create a standard curve for relative quantification of PCR amplicons, one of three different accepted scientific methods for real-time PCR [31] that have been previously published [7,9,28]. To correct for procedural variability, cyclophilin (housekeeping gene) from each test sample was used to normalize target amplicons. Relative values of FGF2, Ang1, leptin, AdipoR2, and cyclophilin were quantitated from the relative standard curve. Values were then transformed to log_10_ and normalized with cyclophilin. Cultured cell values are represented as a percentage of the control (0 μg, no adiponectin treatment). Agarose gel electrophoresis, containing ethidium bromide, was used to confirm the presence of a single band of the expected size of the PCR amplicon for each of the PCR primer pair reactions.

### 2.5. Dispersed Corpora Lutea Cell Cultures

Luteal tissue was processed as previously reported [4,7]. Briefly, tissue was mechanically minced and enzymatically dissociated in digestion media (Type IA collagenase 1.27 mg/mL, 0.5% bovine serum albumin (BSA) in Dulbecco’s Modified Eagle’s Medium (DMEM); Sigma Aldrich) for 90 min at 37 °C with gentle agitation in a shaking water bath. Cells were filtered through a metal mesh (80 μm) and centrifuged twice at 150× *g* for 4 min to form a pellet to remove the enzymatic supernatant. Cells were re-suspended in DMEM, and the numbers of viable cells were determined using the Trypan Blue Exclusion Test; viability was >85% for all cell cultures. The dispersed lutea cells were seeded at 3 × 10^6^ cells/culture well (24-well polystyrene plate, Sigma-Aldrich) in attachment serum media (1 mL/well; DMEM supplemented with 10% (*v*/*v*) heat-inactivated charcoal stripped fetal bovine serum (CS-FBS; Avantor/VWR) and 5000 μg/mL penicillin/streptomycin (Sigma-Aldrich)) and incubated for 24 h in a humidified atmosphere of 5% CO_2_ in air at 37 °C to equilibrate the cells to the culture condition, allow aggregation, and promote adherence. Following the 24 h incubation, attachment serum media were aspirated and replaced with culture media (1 mL/well; DMEM, 0.1% (*w*/*v*) BSA, 2.0% (*v*/*v*) CS-FBS, 0.5 mM ascorbic acid, and 5000 μg/mL penicillin/streptomycin) containing recombinant porcine adiponectin (RD572023100, BioVender, Candler NC) at 1 μg, 3 μg, 6 μg (*n* = 3 wells/dose), or without adiponectin, 0 μg (control; *n* = 3 wells), for 24 h in a humidified atmosphere of 5% CO_2_ in air at 37 °C. The range in culture doses of adiponectin was based on reported follicular fluid concentrations of adiponectin [36], plasma concentrations [25], and adiponectin-cultured endothelial and ovarian cells [16,26]. The inclusion of CS-FBS in the culture media provides an environment that enhances cell viability and supports angiogenic-related processes [37]. After 24 h, culture media was aspirated and stored at −80 °C for the quantitation of secreted progesterone as a marker for cell responsiveness to adiponectin treatment in vitro [26]. A denaturing solution (1 mL/well; 25 mM sodium citrate (pH 7.0), 0.5% (*w*/*v*) *N*-laurylsarcosine, 4 M guanidine thiocyanate with 0.7% (*v*/*v*) 2-mercaptoethanol) was added to cells, aspirated, pooled by dose, and stored at −80 °C for total RNA extraction and mRNA content analysis via real-time PCR.

### 2.6. Radioimmunoassay

Serum and culture media were analyzed for progesterone concentration by radioimmunoassay (RIA, Coat-A-Count^®^; Diagnostics Product Corporation, Los Angeles, CA, USA) to confirm cell viability during the culture process, response to culture treatment, and the presence of a functional CL at the time of tissue harvest. Utilization of the RIA kit was previously validated for porcine serum and culture media [4,7,38,39]. Inter- and intra-assay coefficients of variation for all assays were <10%. All samples were determined in triplicate as has been previously reported [4,7].

### 2.7. Statistical Analyses

Progesterone concentrations, tissue gene expression, and cellular gene expression were analyzed using the MIXED model procedure of SAS as has been previously reported for similar experimental designs [4,7,9,28,40,41]. For tissue expression analysis, the day of the estrous cycle was the fixed source of variation, and the sow was the random source of variation. The adiponectin treatment dose was the fixed source of variation for gene expression analysis in primary cell cultures, and the sow was the random source of variation in the mixed model. For serum progesterone concentrations, the day of the estrous cycle was the fixed source of variation, and the sow was the random source of variation. For the analysis of secreted concentrations of progesterone in culture media, the dose of adiponectin treatment was the fixed source of variation, and the sow was the random source. Mean comparisons were determined using the PDIFF procedure of SAS when a significance of *p* < 0.05 of the mixed model was detected. All values are represented as the least-squares mean plus or minus the standard error of the mean (LSmeans ± SEM).

## 3. Results

### 3.1. Progesterone Concentrations in Serum and Cultured Media

**Figure 1:**
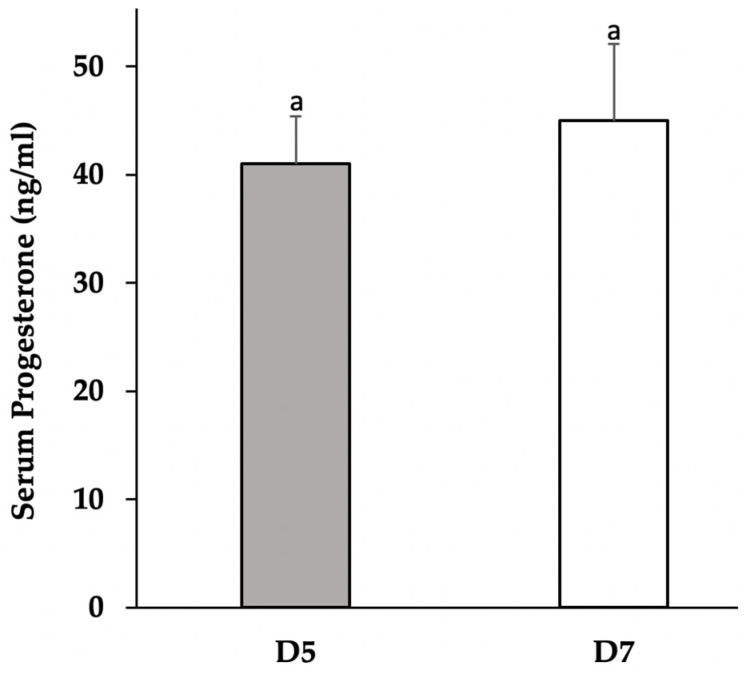
Serum concentrations of progesterone from sows collected on D5 (*n* = 4) and D7 (*n* = 5) of the estrous cycle. Progesterone did not significantly differ (^a^ *p* > 0.05) between days.

**Figure 2:**
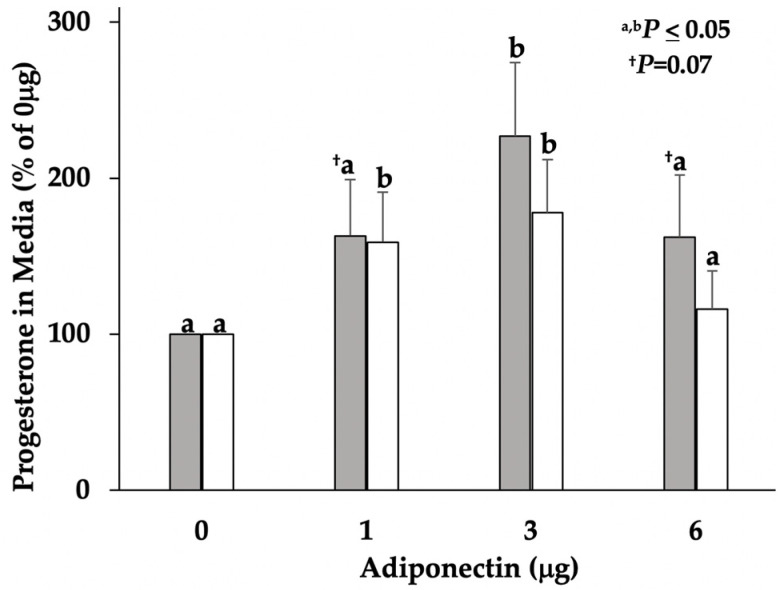
Concentrations of progesterone in aspirated culture media from D5 (shaded bars; *n* = 4) and D7 (open bars; *n* = 5) dispersed lutea cell cultures, following 24 h treatment with adiponectin (1, 3, and 6 μg). Adiponectin treatment significantly increased (*p* ≤ 0.05) secreted progesterone in D5 and D7 cultures in a bell-shaped dose response. ^a,b^ Different superscripts identify means relative to treatment dose of adiponectin, within day, that significantly differ (*p* < 0.05) from control dose (0 μg; no adiponectin). ^†^ Superscript identifies mean concentrations of progesterone relative to treatment dose of adiponectin, within day, that tended (*p* = 0.07) to differ from control dose (0 μg).

Elevated concentrations of progesterone were detected in serum collected from sows on D5 and D7 of the estrous cycle, demonstrating the presence of functional ovarian luteal tissue. Mean concentrations of serum progesterone were numerically different between D7 (44.9 ± 7.1 ng/mL) and D5 sows (41.6 ± 4.4 ng/mL) but were not significant (Figure 1). Media progesterone concentrations increased (*p* ≤ 0.05; Figure 2) in a bell-shaped dose response in both D5 and D7 cultures, confirming a cellular response to adiponectin.

### 3.2. Gene Expression of FGF2, Ang1, Leptin, and AdipoR2 in Tissue and Cell Cultures

**Figure 3:**
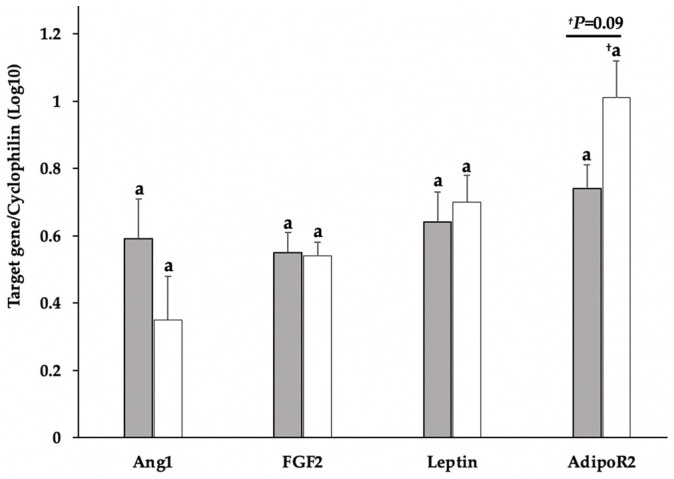
Gene expression of Ang1, FGF2, leptin, and AdipoR2 in luteal tissue from sows on D5 (shaded bars; *n* = 4) and D7 (open bars; *n* = 5) of the estrous cycle. Relative quantities of amplicons were transformed to log_10_ and normalized with cyclophillin. ^a^ Superscript identifies means that did not statistically differ (^a^ *p* > 0.05). ^†^ Superscript identify means that tended (^†^ *p* = 0.09) to differ relative to day.

**Figure 4:**
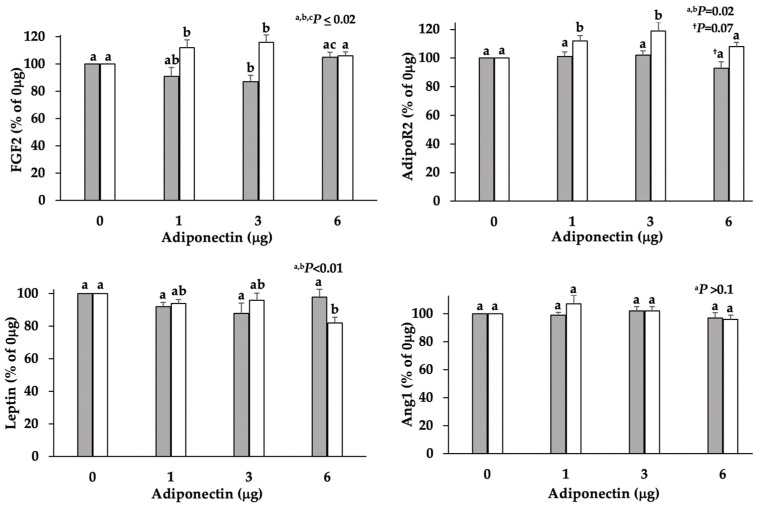
Gene expression of Ang1, FGF2, leptin, and AdipoR2 in D5 (shaded bars; *n* = 4) and D7 (open bars; *n* = 5) dispersed lutea cell cultures following 24 h treatment with adiponectin (1, 3, and 6 μg). Relative quantities of amplicons were transformed to log_10_, normalized with cyclophilin, and expressed as a percentage of the control dose (0 μg, no adiponectin). ^a,b,c^ Different superscripts identify means that significantly differ (*p* < 0.05) within day relative to dose of adiponectin. ^†^ Superscript identifies means that tended (*p* = 0.07) to differ relative to dose of adiponectin within day.

Luteal content of leptin, FGF2, and Ang1 mRNA did not differ relative to D5 or D7 of the estrous cycle; however, AdipoR2 tended (*p* = 0.09; Figure 3) to be higher in D7 tissue. In adiponectin-treated D5 dispersed lutea cell cultures, FGF2 mRNA content decreased (*p* ≤ 0.02; Figure 4) in an inverse, bell-shaped dose response. Of interest is the adiponectin-dependent, bell-shaped, dose-responsive increase (*p* < 0.01, Figure 4) in FGF2 mRNA that occurred in the D7 cells. Adiponectin also induced (*p* = 0.02, Figure 4) a bell-shaped, dose response similar to FGF2 in AdipoR2 expression in the D7 cultures. In contrast to D7, AdipoR2 merely tended (*p* = 0.07) to decrease in D5 cells treated with the highest dose (6 μg) of adiponectin. Adiponectin did not significantly influence leptin in D5 cells; nevertheless, leptin decreased in D7 cells with a significant (*p* < 0.01; Figure 4) mean separation occurring in the highest dose (6 μg) of adiponectin. Adiponectin did not appear to influence Ang1 expression in either D5 or D7 cultures. 

### 3.3. Immunohistochemistry of AdipoR2 in the CL

**Figure 5:**
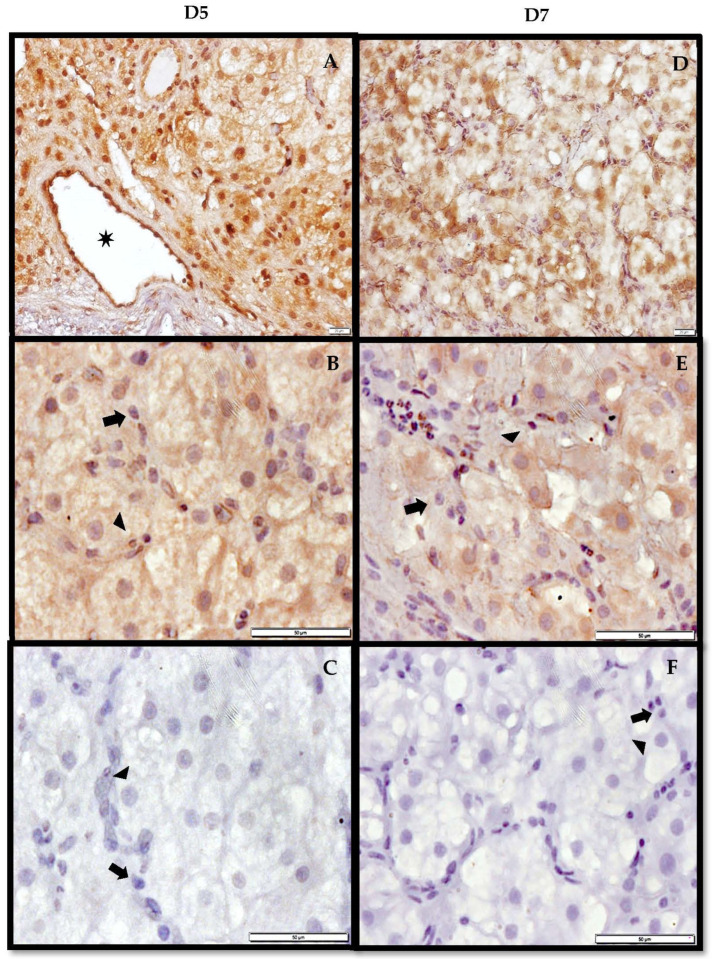
Representative immunohistochemistry of AdipoR2 in a D5 (**A**–**C**) and D7 (**D**–**F**) CL using NovaRED^®^ (Vector Laboratories) and stained with hematoxylin. Panels A and D are 20× magnification of the tissue section. Panels B and E are 40× magnification of tissue sections A and D, respectively. Panels C and F are 40× magnification of tissue sections incubated in normal serum. Star indicates vessel, black arrow points to small luteal cells, and black arrowhead points to large luteal cells. Scale bar is 20 μm in 20× magnification panels and 50 μm in 40× magnification panels.

Adiponectin receptor 2 was detected in both D5 and D7 luteal tissue with evident spatial variations relative to the day (Figure 5). In D5 tissue, AdipoR2 was predominantly localized along the lumen of vessels and the cytosol of both large and small luteal cells. To a lesser extent, the receptor was also present on the cell membrane of small and large luteal cells. In contrast to D5, D7 tissue exhibited an opposite staining pattern where cell membrane localization of AdipoR2 in both large and small luteal cells was more prevalent and distinct. The receptor was also present along the lumen of vessels and in the cytosol of both large and small luteal cells, but less prominent than in D5 tissue.

## 4. Discussion

As previously reported [4], leptin and Ang1 did not differ in expression in CL tissue on either D5 or D7 of the estrous cycle in domestic, mature female pigs. In contrast to leptin and Ang1, FGF2 did not reflect a previous report of higher luteal FGF2 mRNA content on D7 than on D5 [4]. However, Katchko et al. [4] observed that the expression level of FGF2 was changed in a fourth-degree polynomial curve, i.e., high on D4 and 6 and lower on days D5 and D7. Therefore, the expression of the tissue FGF2 reported herein is not considered to be anomalous. Interestingly, the expression of AdipoR2 tended to be higher on D7 than on D5, indicating that adiponectin-mediated action may be involved in the developmental process. Luteal adiponectin mRNA has been detected in the early (D2–3), mature (D10–12), and regressing porcine CL (D14–16), but protein content appears to be highest in the early stage [10]. A similar mRNA and protein pattern was observed for both AdipoR1 and AdipoR2 [25]. However, with respect to adiponectin-mediated action on angiogenic processes, AdipoR2 facilitates the vascularization of tissue, as demonstrated in AdipoR2-KO mice compared to the AdipoR1-KO [20]. Furthermore, AdipoR2 expression has been detected in both endothelial progenitor cells [42] and vascular endothelial cells [43]. In the study herein, AdipoR2 was localized to the lumen of vessels in both D5 and D7 CL, implicating a potential role for adiponectin in luteal vasculature. In addition to the vasculature, AdipoR2 was also localized to the cytoplasm and cell surface of small and large luteal cells with a more prevalent spatial distribution along the cell surface in D7 tissue. Luteal cells are the steroidogenically capable components of luteal tissue that produce and maintain concentrations of progesterone required for gestational support. The presence of AdipoR2 in luteal cells suggests that adiponectin may influence steroidogenic processes. This is supported by the increase in progesterone secretion in the D5 and D7 cultures, but this response appears to contradict previous reports. Gupta et al. [26] demonstrated that adiponectin reduces progesterone secretion and the expression of steroidogenic enzymes in cultured bovine mid-luteal cells sourced from an abattoir. Similar results were reported in porcine luteal cells collected from estrus-monitored sows on D10–12 of the estrous cycle [10]. However, Smolinska et al. [44] demonstrated that adiponectin increased steroidogenic enzymes in porcine myometrial explants and progesterone secretion from endometrial and myometrial explant cultures. Such variation may be attributed to culture conditions, such as serum-free vs. serum-inclusive media, and/or the stage of the luteal phase from which cells were obtained for culture. In the luteal cell culture studies, serum was not a component of the treatment media, but it was a component in the uterine explant cultures and in the study herein. The serum contains insulin-like growth factors (IGF) [45], and the addition of IGF-I to adiponectin-treated human and bovine luteinized granulosa cells stimulates rather than inhibits progesterone secretion [46,47]. Although the culture media in the current study used CS-FBS, it still contains measurable quantities of IGF, albeit lower than in FBS [45], and may explain the effect of adiponectin on secreted progesterone. Nevertheless, the age of the CL should also be taken into consideration given that adiponectin and adiponectin receptor luteal protein content is highest in the early stage [10,25]. This is a stage when tissue mass is accruing, angiogenic activity is high, and progesterone secretion is steadily increasing [1], all of which may influence luteal tissue response to an adipokine. In support, the adipokine leptin inhibits progesterone secretion in cultured dispersed caprine lutea harvested on D3 of the estrous cycle, but this response is attenuated in D10 and abrogated in D15 cultures [7]. Further evidence of an age-associated response to adipokine is the inverse expression of FGF2 in adiponectin-treated dispersed lutea cell cultures reported herein, despite stimulation in progesterone secretion in both D5 and D7 cultures. The role of FGF2 in luteal development has predominantly been associated with angiogenic processes, including endothelial cell proliferation, migration, and capillary network formation [1]. It has been reported that the expression of FGF2 changes daily in the developing porcine CL [4], which may be associated with the temporal sensitivity of endothelial cells to FGF2-mediated action [48,49]. While this temporal sensitivity would not fully explain the day-dependent differential response to adiponectin, the availability of AdipoR2 for ligand-binding could contribute to the observed variation. Tian et al. [50] demonstrated that the overexpression of AdipoR2 enhances cellular response in AdipoR1-deficient monocytes compared to monocytes expressing normal quantities of AdipoR2. The immunohistochemical staining for AdipoR2 was more prevalent at the surface of large and small luteal cells in D7 than in D5 tissue, indicating that luteal AdipoR2 is likely more available for adiponectin binding and subsequent cellular response on D7. Further supporting the role of AdipoR2 in mediating adiponectin action in the D7 cells is the expression of AdipoR2 that mimicked the dose–response pattern observed for FGF2 in D7 cells. This positive regulatory relationship between adiponectin and AdipoR2 has also been observed in muscle tissue and cancerous prostate cells [51,52]. However, this does not explicate the decrease in FGF2 in D5 cultures. The occurrence of cell-surface AdipoR2 is less prevalent than the cytosolic localization in D5 tissue, implying that there is less AdipoR2 available for ligand binding and reduced potential for adiponectin-mediated action. Nevertheless, it is conceivable that D5 effects are mediated through AdipoR1, which is also expressed in the early-stage porcine CL [25]. Although plausible, it is perplexing in that the receptor isoform typically influences the magnitude of the response in the expression of cytokines and growth factors rather than opposing the action [50,53]. This was demonstrated in leptin expression where it decreased in D7 cells but was not significantly reduced in D5 cultures. The effect of adiponectin on leptin expression is remarkably limited, and instead, adiponectin appears to influence leptin-mediated affects by antagonizing leptin activity [54,55], in part by abrogating the signal transduction capacity of its receptor [56,57,58]. Nevertheless, Jarde et al. [59] reported a tendency towards a reduction in leptin expression in adiponectin-treated MCF-7 breast cancer cells. Unlike leptin and FGF2, adiponectin did not significantly influence Ang1 expression, despite reported adiponectin-mediated action in luteinized granulosa cells through the PPARα signaling pathway [60], a mechanism that regulates angiopoietins in endothelial cells [23]. Collectively, adiponectin plays a functional role in the developing porcine CL associated with AdipoR2 that is related, in part, to the regulation of pro-angiogenic factors.

## 5. Conclusions

Adiponectin is a complex adipokine that elicits numerous biological actions through unconventional receptors. Some of these actions include the regulation of processes important to reproduction including the secretion of progesterone, which is critical for pregnancy maintenance. More importantly, adiponectin may also be involved in luteal angiogenesis, a process essential for the transport of substrates needed to support the rapidly developing CL. Its receptor, AdipoR2, is localized to luteal vasculature and luteal cells, which are known to express pro-angiogenic factors. Furthermore, adiponectin influences the expression of FGF2, an important regulator of luteal vascular development, and the adipokine leptin, which also regulates luteal FGF2. Future research will further elucidate the role of adiponectin in luteal angiogenic processes.

## Figures and Tables

**Table 1:** Primer sequences for real-time PCR.

Gene	Forward 5′→3′	Reverse 5′→3′	Anneal	Accession No.	Ref.
AdipoR2	CCATTCTCTGCCTTTCTTTTTCG	CTGCTCTTACTCCCCGATACTGA	58 °C	AY452711	[32]
Ang1	GAGCAGCCTGATCTTACATG	GCATTCTCTGTAGTCAAGCC	53 °C	NM213959	[7]
FGF2	TCAAAGGAGTGTGTGCGAAC	CAGGGCCACATACCAACTG	55 °C	AJ577089	[33]
Leptin	ACAGAGGGTCACCGGTTTGG	TAGAGGGAGGCTTCCAGGAC	61 °C	AF0226976	[34]
Cyclophilin	TGCCATCCAACCACTCAG	TAACCCCACCGTCTTCTT	52 °C	AF14571	[35]

## Data Availability

The data reported herein are available upon request from the corresponding author.
